# Prognostic value of orthogeriatric assessment parameters on mortality: a 2-year follow-up

**DOI:** 10.1007/s00068-021-01727-8

**Published:** 2021-06-25

**Authors:** Andreas Wiedl, Stefan Förch, Annabel Fenwick, Edgar Mayr

**Affiliations:** Universitätsklinikum Augsburg, Abteilung Für Unfallchirurgie, Orthopädie, Plastische und Handchirurgie, Stenglinstraße 2, 86156 Augsburg, Germany

**Keywords:** Orthogeriatric assessment, Mortality, Parker-Mobility Score, Barthel Index, Place of residence, Care level, Length of stay

## Abstract

**Introduction:**

Since the arise of orthogeriatric co-management patients’ outcome and survival has improved. There are several assessment parameters that screen the precondition of orthogeriatric patients including mobility, activities of daily living, comorbidities, place of residence and need for care just to name a few. In a 2-year follow-up on an orthogeriatric co-managed ward the fracture-independent predictive value of typical assessment parameters and comorbidities on the associated mortality was examined.

**Methods:**

All patients treated on an orthogeriatric co-managed ward from February 2014 to January 2015 were included. No fracture entity was preferred. Emphasis was set on following parameters: age, gender, Parker-Mobility Score (PMS), Barthel Index (BI), Charlson-Comorbidity Index (CCI), dementia, depression, sarcopenia, frequent falling, length of stay (LOS), care level (CL) and place of residence (POR). In a 2-year follow-up the patients’ death rates were acquired. SPSS (IBM Corp., Armonk, New York, USA) and Cox regression was used to univariately analyze the expression of the mentioned parameters and mortality course over 2 years from discharge. In a multivariate analysis intercorrelations and independent relationships were examined.

**Results:**

A follow-up rate of 79.6% by assessing 661 patients was achieved. In the univariate analysis linear inverse correlation between PMS and BI and mortality and a linear positive correlation between CCI and higher mortality were observed. There was also a significant relationship between lower survival and age, dementia, sarcopenia, frequent falling, higher institutionalized place of residence and higher CL. No univariate correlation between 2-year mortality and gender, depression and LOS was found. In the multivariate Cox regression, the only independent risk factors remaining were lower PMS (HR: 1.81; 95%CI: 1.373–2.397), lower BI (HR: 1.64; 95%CI: 1.180–2.290) and higher age per year (HR: 1.04; 95%CI: 1.004–1.067).

**Conclusion:**

Age, PMS, BI, CCI, preexisting dementia, sarcopenia, frequent falling, POR and CL are univariate predictors of survival in the orthogeriatric context. An independency could only be found for PMS, BI and age in our multivariate model. This underlines the importance of preexisting mobility and capability of self-support for the patient’s outcome in terms of survival.

## Introduction

With the increasing burden of fragility fractures [1], concepts of orthogeriatric co-management were established in the clinical treatment to address the injured old patient more multidimensionally [2].

Since the implementation of co-managed orthogeriatric treatment there is plenty evidence showing improved associated outcome for patients suffering from fragility fractures, especially hip fractures [3–5]. Rarely studies analyze more diverse cohorts of orthogeriatric patients that suffer from different or multiple injuries [6, 7]. Several assessment and outcome parameters were evaluated and confirmed as “a standard set” in the scholar examination of orthogeriatric co-management of hip fractures [8]. Amongst those are mobility, activities of daily living, living situation (nursing home or private home), comorbidities, cognitive status, and length of stay (LOS). There is research on the predictive value of the mentioned assessment parameters and short and long term mortality after hip fractures [4, 5, 9, 10]. Charlson-Comorbidity Index (CCI), American Society of Anesthesiologists (ASA) Score, Parker-Mobility Score (PMS), Barthel Index (BI), place of residence (POR) prior to admission, frailty, need for care and preexisting dementia have been approved as independent risk factors in different settings [4, 5, 11–13]. These risk factors are in most cases just partly included in analyses. The importance and intercorrelation of orthogeriatric assessment parameters and other premorbid conditions on mortality in the diversity of an orthogeriatric cohort that suffers from different fragility fractures like humeral, vertebral, femoral or others (e.g., forearm, pelvic, ankle joint, etc.) has yet to be examined.

The goal of this investigation is to determine the prognostic value of orthogeriatric assessment parameters on the mortality rate of inward patients suffering from different fragility fractures on an orthogeriatric co-managed ward in a 2-year follow-up. Being a fracture-independent stratification, this shall serve as a general overview concerning these parameters on mortality in the orthogeriatric context.

## Methods

All patients treated on an orthogeriatric co-managed ward from February 2014 to January 2015 were assessed. Informed consent of patients, respective their legal guardians was achieved. There was a positive approval of the Bavarian state chamber of medicine on the performance of the study (Sign: 7/11192). Patients were admitted to the orthogeriatric ward if they met several criteria that were counted as a score: age over 75 with typical comorbidities like impaired mobility and the need for aids, dementia, acoustic and visual impairments, polypharmacy, sarcopenia or frequent falling. Every item was taken into account for 1 scoring point, except impaired mobility which counted as 2. Reaching at least 4 points qualified for admission. General exclusion criteria were advanced dementia and immobility. No specific injury or cause for admission was preferred. Pathologic fractures, need for revision surgery and infections were included as well as primary trauma. Atraumatic bony tumor diseases were not included.

Primary assessment variables were reason for admission and typical orthogeriatric parameters as mentioned below that have been examined through geriatric assessment and the respective medical history. Ranked Subgroups were formed for each parameter to enable comparison in mortality and determine differences. A listing of the groups with their specific cutoffs is displayed in Table 2.

### Mobility, activities of daily living (ADL) and comorbidities

Typical geriatric assessment parameters were chosen in the determination of functional status and comorbidities. The Parker-Mobility Score (PMS) prior to the debilitating trauma was achieved and patients were split into three groups: PMS 0–3, PMS 4–6 and PMS 7–9.

ADL were assessed by Barthel Index (BI) after completion of the stationary treatment right before discharge, here again three groups were formed: BI 0–30, BI 35–65 and BI 70–100.

The comorbidities were determined in the medical history on admission and the respective CCI was calculated, dividing patients in the groups CCI 0–1, CCI 2–3 and CCI ≥ 4.

### Cognition and depression

Cognitive status was assessed on admission either by known dementia or by Mini-Mental-Status Examination (MMSE). As literature suggests, a cutoff of 24 score points was used to subdivide into demented and not demented, labelling every result with less than 24 score points as demented and higher results as not demented [14]. The geriatric depression scale (GDS) was used to display the distribution of prevailing depression, setting a cutoff point of 5. Every result with at most 5 points labelled as not depressed, every result higher as depressed [15].

### Sarcopenia and frequent falling

Sarcopenia on admission was determined by the patients’ respective maximal calf circumference measurements using a cutoff value of 33 cm [16]. Every calf-circumference being at least 33 cm labelled the patient as not sarcopenic, vice versa as sarcopenic with the calf-circumference being lower.

Falls frequency was assessed by the medical history, if there were at least two falls in 6 months prior to admission the according patient was grouped as a frequent faller.

### Care level (CL), place of residence (POR) and length of stay (LOS)

The previous POR was determined on every admission, discriminating between private home (PV), assisted living (AL) and nursing home (NH).

The existing CL was recorded and used as an indirect indicator of the need for care and reduced ADL. Depending on the national provisions during the assessment period, there were 3 CL’s splitting patients into four groups: no CL at all, CL 1, CL 2 and CL 3. The categorization to the respective CL was dependent on the frequency of daily support needed and daily hours spent for care by caregivers [17].

The LOS was calculated and is represented in the three groups LOS < 1 week, LOS 1–2 weeks and LOS > 2 weeks. LOS itself was examined for its dependency on the respective discharge accommodation of previously home-dwelling patients.

### Treatment

Orthogeriatric co-management was provided for every patient. Next to being treated by both an orthopedic and geriatric specialist, patients attended daily physio- (twice) and ergotherapy. Fractures of the lower limb were generally treated surgically to provide early mobilisation. Humeral and forearm fractures were mostly addressed operatively (87% of cases of follow-up cohort). Vertebral fractures underwent a decisional algorithm taking into account fracture morphology, clinical course, pain and course of radiographic changes resulting in a fifty–fifty ratio of surgical to conservative treatment.

Pathologic fractures were treated according to the patient’s respective prognosis either by internal fixation or prosthesis and if possible, with combined radiation therapy.

Soft tissue infections were treated by incision and debridement, prosthetic infections were treated by revision of prosthesis and either total or partial exchange depending on infection’s severity and acuity.

Pelvic and rib fractures were treated conservatively.

### Follow-up

Follow-up was generated after 2 years by sending questionnaires to patients and relatives determining survival. In case of receiving no response by mail, a maximum of 5 attempts of contact via phone call was performed. Should the respective patient having been deceased, the exact month of death was inquired. Patients that were lost to follow up were excluded.

### Data analysis

SPSS (IBM Corp., Armonk, New York, USA) was used for data analysis. Mean values and standard deviations were determined for continuous variables. *T* tests for independent samples were used for comparison of mean values. Mortality differences between the variable groups were evaluated by Fisher’s exact test and log-rank test for their significance. A Cox-proportional model was set up to adjust the according mortality risks of assessment parameters in a uni- and multivariate analyses. To provide a valid analysis at least ten events per independent variable had to be obtained [18].

## Results

A total of 830 patients were initially assessed collecting follow-up data from 661 (79.6% follow-up rate), consisting of 165 men and 496 women. The average age was 84.6 years. Table 1 shows general parameters as well as the respective reasons for admission. The main contributing injury concerned the lower extremity followed by fractures of the upper extremity, spine and multiple injuries. Orthogeriatric scores and assessment parameters could not be determined in each case, the actual assessment rate of each variate and the distribution of each parameter is stated in Table 2.

**Table 1:** Mean age, function and mobility, gender and injury distribution

	Follow-up cohort
Age (years)	84.6 ± 6.57
Gender	
Male	165
Female	496
Injuries	
Upper extremity	97
Lower extremity	306
Spine	85
Pelvis	34
Ribs	19
Infections	15
Multiple injuries	44
Other injuries/causes of admission	61
PMS before admission	5.3 ± 2.61
BI on discharge	43.2 ± 19.89

**Table 2:** Assessment rate and distribution of assessment variables

Assessment parameters	Assessment rate	Ranked groups	Distribution
Parker-Mobility Score (PMS)	85.3% (564)	PMS 7–9	186
		PMS 4–6	204
		PMS 0–3	174
Barthel Index (BI)	85.3% (564)	BI 70–100	63
		BI 35–65	337
		BI 0–30	164
Charlson-Comorbidity Index (CCI)	97.7% (664)	CCI 0–1	168
		CCI 2–3	239
		CCI ≥ 4	239
Dementia by Mini-Mental-Status Examination (MMSE)	83.1%(549)	MMSE ≥ 24	343
		MMSE < 24	206
Depression by Geriatric Depression Scale (GDS)	79.7% (527)	GDS ≤ 5	386
		GDS > 5	141
Sarcopenia by calf circumference	83.2% (550)	< 33 cm	229
		≥ 33 cm	321
Frequent falling by falls during the previous 6 months	97.4% (644)	≥ 2	482
		< 2	162
Length of stay (LOS)	100% (661)	< 1 week	81
		1–2 weeks	249
		> 2 weeks	331
Care Level (CL)	95.3% (630)	No CL	326
		CL 1	203
		CL 2	89
		CL 3	12
Place of residence on admission (POR)	96.7% (639)	Private home (PH)	470
		Assisted living (AL)	38
		Nursing home (NH)	131

Stratified 1- and 2-year mortalities and according *p* values are listed in Table 3, the univariate analysis of respective HR’s is stated in Table 4.

**Table 3:** 1- and 2-year mortality stratified for assessment parameters and *p* values

	1-year mortality		2-year mortality	
		*p* value		*p* value
Age (years)				
71–80	14.9%	*p* = 0.000	26.4%	*p* = 0.000
81–90	27.9%		38.9%	
91–95	39.4%		55%	
Gender				
Male	34.5%	*p* = 0.015	41.8%	*p* = 0.31
Female	24.4%		37.3%	
PMS				
PMS 7–9	13.4%	*p* = 0.000	18.3%	*p* = 0.000
PMS 4–6	23.0%		35.3%	
PMS 0–3	39.7%		53.4%	
BI				
BI 70–100	9.5%	*p* = 0.000	12.7%	*p* = 0.000
BI 35–65	19.6%		32.0%	
BI 0–30	43.3%		54.9%	
CCI				
CCI 0–1	19.0%	*p* = 0.007	26.8%	*p* = 0.000
CCI 2–3	26.8%		39.7%	
CCI ≥ 4	33.1%		46.0%	
Dementia				
No dementia	21.0%	*p* = 0.000	31.8%	*p* = 0.000
Dementia	37.4%		51.5%	
Depression by GDS				
GDS ≤ 5	22.0%	*p* = 0.246	31.9%	*p* = 0.176
GDS > 5	27.0%		38.3%	
Sarcopenia				
No	19.6%	*p* = 0.000	29.0%	*p* = 0.000
Yes	38.3%		45.9%	
Frequent falling				
No	21.0%	*p* = 0.065	27.2%	*p* = 0.001
Yes	28.6%		42.1%	
LOS				
< 1 week	33.3%	*p* = 0.319	42.0%	*p* = 0.583
1–2 weeks	27.3%		36.1%	
> 2 weeks	25.1%		39.3%	
CL				
No CL	18.1%	*p* = 0.000	25.8%	*p* = 0.000
CL 1	33.5%		47.8%	
CL 2	29.2%		40.4%	
CL 3	50.0%		75.0%	
POR				
PV	23.2%	*p* = 0.000	32.3%	*p* = 0.000
AL	21.1%		44.7%	
NH	40.5%		56.5%

**Table 4:** Univariate analysis and corresponding HR for each risk factor

Risk factor	*p*	HR (95%CI in Brackets)
Age	0.000	1.05 (1.031–1.073)
Gender	0.133	0.81 (0.613–1.067)
PMS	0.000	1.91 (1.586–2.297)
BI	0.000	2.31 (1.822–2.933)
CCI	0.000	1.36 (1.156–1.598)
Dementia	0.000	1.94 (1.481–2.530)
Depression	0.172	1.25 (0.907–1.721)
Sarcopenia	0.000	1.80 (1.358–2.375)
Frequent falling	0.001	1.70 (1.224–2.350)
LOS	0.743	0.97 (0.811–1.161)
CL	0.000	1.46 (1.258–1.698)
POR	0.000	1.45 (1.258–1.660)

**Fig. 1:**
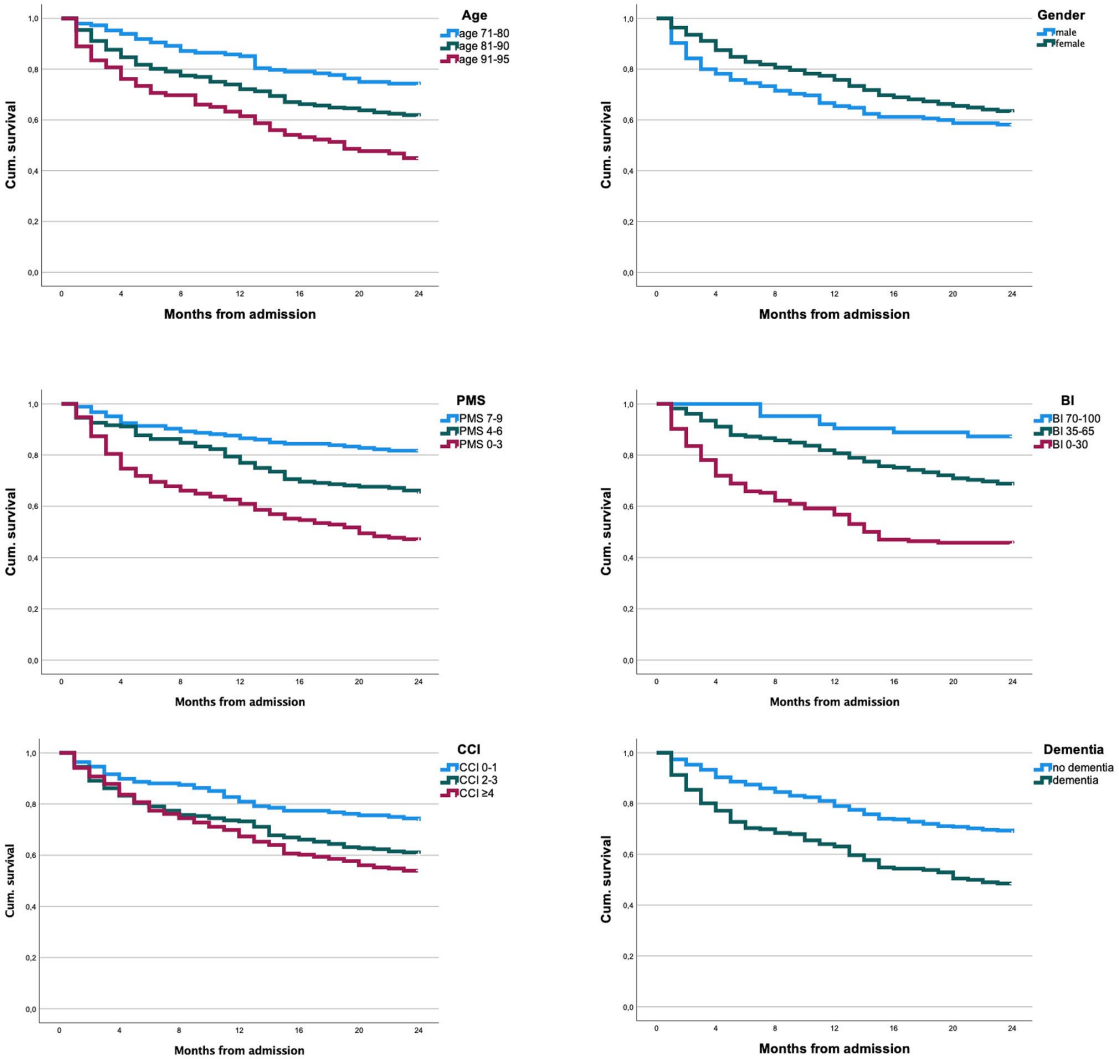
Survival curves, respectively, stratified for age, gender, PMS, BI, CCI and dementia

**Fig. 2:**
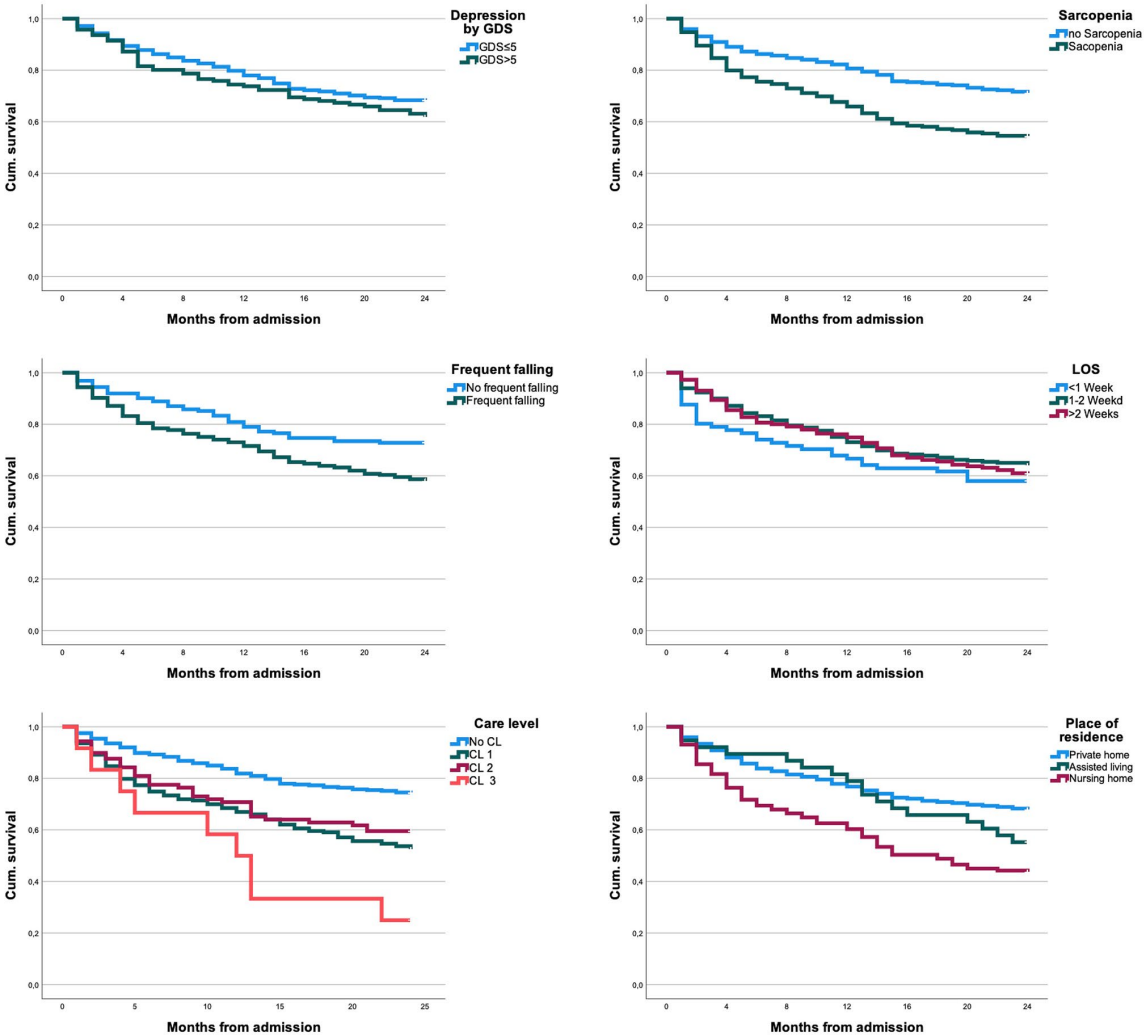
Survival curves, respectively, stratified for depression, sarcopenia, frequent falling, LOS, care level and place of residence

Figures 1 and 2 display Kaplan–Meier curves associated to the risk factors.

### Age and gender

Comparing age groups 71–80, 81–90 and 91–95 years in both 1- and 2-year mortality a linear increase in mortality could be observed (*p* < 0.001). Gender shows a lower 1-year mortality for female patients (*p* = 0.015). This difference vanishes after 2 years showing death rates of 41.8% for men and of 37.3% for women (*p* = 0.31).

### Function, mobility, comorbidities

PMS and BI display inverse linear correlations between mobility, respective function and mortality (Fig. 1). The CCI also shows stepwise reduced survival with more existing comorbidities. Every association shows statistical significance (*p* < 0.05). 2-year mortality in the respective best and weakest groups is as following for PMS: 18.3%: 53.4%, BI: 12.7%: 54.9% and CCI: 26.8%: 46.0%.

### Cognition and depression

Preexisting dementia significantly correlated with increased mortality. 2-year mortality was 31.8% for cognitively capable patients, 51.5% for patients with dementia (*p* = 0.000). Existing depression as screened by GDS showed no significant influence on mortality (2-year mortality: 31.9% for GDS ≤ 5, 38.3% for GDS > 5, *p* = 0.176).

### Sarcopenia and frequent falling

Sarcopenia showed a significant relation with increased mortality, displaying a 2-year mortality of 45.9% compared to 29.0% for non-sarcopenic patients (*p* = 0.000). Frequent fallers had lower survival rates in comparison to non-frequent fallers, showing significance after 2 years of observation (mortality rates after 2 years: 27.2%: 42.1%, *p* = 0.001).

### LOS, CL and POR

No association between mortality and LOS was seen in the cohort. Patients who were discharged in the first week showed a tendency to the highest death rates after 1 year (33.3%) and after 2 years (42.0%). LOS was dependent on the respective discharge accommodation. It was significantly raised in patients that lived at home and were discharged to rehabilitation (18.4 days; *n* = 207; *p* < 0.001) or to a nursing home (14.8 days; *n* = 51; *p* = 0.048) compared to patients that were discharged to their homes (12 days; *n* = 193).

The existing CL showed a significant correlation between higher need for care and death rates. Although mortality for CL 1 and 2 was comparable, the difference between no existing CL and CL 3 was remarkable (2-year mortality for no CL: 25.8%, compared to CL 3: 75.0%; *p* = 0.000).

Correlations were also found for preexisting POR, showing the best outcome for patients that lived at home prior to admission. Living in a nursing home was associated to the highest death rates (2-year mortality for patients from a PV: 32.2%; for patients having lived in a NH: 56.5%, *p* = 0.000).

### Interferences and independent correlations

A multivariate Cox-regression was performed being displayed in Table 5. Both GDS and LOS were removed from the model, not showing significant impact in the performed univariate analysis. After correction 381 cases (130 events) with overall complete assessments in the ten involved risk factors remained. The strongest independent correlation demonstrated PMS with a stepwise HR of 1.81 (95%CI: 1.373–2.397) for more immobile patients. BI showed a significant HR of 1.64 (95%CI: 1.180–2.290) for cohorts capable of less ADL. Age also persisted as an independent factor displaying a HR of 1.04 (95%CI: 1.004–1.067) per year. Every other parameter did not remain as a significant influential factor. Higher CL, more institutionalized POR and frequent falling even insignificantly correlated with improved survival after correction for cofactors.

**Table 5:** Multivariate analysis and corresponding HR’s

Risk factor	*p*	HR (95%CI in brackets)
Age	0.025	1.04 (1.004–1.067)
Gender	0.211	0.78 (0.523–1.154)
PMS	0.000	1.81 (1.373–2.397)
BI	0.003	1.64 (1.180–2.290)
CCI	0.178	1.18 (0.929–1.490)
Dementia	0.363	1.19 (0.819–1.724)
Sarcopenia	0.238	1.25 (0.865–1.790)
Frequent falling	0.671	0.90 (0.546–1.476)
CL	0.295	0.87 (0.676–1.126)
POR	0.392	0.91 (0.731–1.131)

## Discussion

In this investigation a 2-year follow-up was performed on a variety of orthogeriatric traumatized patients that were treated on a co-managed ward. The emphasis was set on the fracture-independent influence and predictive value of important assessment parameters on patients’ survival rate. The majority of studies that have researched mortality associated risk factors in the orthogeriatric field examined hip fracture cohorts. Rarely a study examines diverse injuries [6]. Having included a follow-up rate of 661 patients the cohort can be seen as adequate for a relevant analysis. Folbert et al. and Schuijt et al. who investigated in according questions had similar cohort strengths [5, 6]. Before comparison to literature is performed, it has again to be taken into account that all patients in this study underwent orthogeriatric treatment and did suffer from a variety of injuries and not one specific fracture.

Age per year and male gender are both reported as significant influencers of higher mortality in general- and hip fractured orthogeriatric patient cohorts [4–6]. As age was also a relevant influential factor in this investigation, we have just seen an insignificant tendency of higher male mortality. Male gender frequently was found to be significantly as well as non-significantly associated to higher mortality after hip fractures [5, 6, 9, 19].

In the three scoring systems PMS, BI and CCI inverse respective linear correlations to mortality have been observed. Lower mobility, lower function and more comorbidities were associated to higher death rates. Originally Parker et al. constructed and evaluated the PMS as a relevant and valid predictive tool for survival in hip fracture patients [20]. Its value as a predictor of mortality risk can again be confirmed in this study, as it has already been for orthogeriatric co-managed hip and non-hip fractures [11, 21]. Lower BI has also been shown to be associated with poorer survival after hip fractures several times being an independent risk factor [5, 22, 23]. Next to age per year, PMS was the strongest independent variate in our multivariate analysis, followed by BI. Any other influential factor that has been proven significantly correlated to mortality in the univariate analysis, revealed dependency in the multivariate analysis. Higher CCI, sarcopenia and dementia were associated with significantly lower survival in their respective univariate analyses. After adjustment only slight insignificant according tendencies remained. We have to suppose interdependencies between those variables that influenced the observed outcome. Knauf et al. showed significant improved 5-year survival after hip fractures for lower CCI, higher BI, and higher MMSE on admission in their multivariate analysis but missing out on other parameters that were integrated in our study (PMS, sarcopenia, frequent falling) [9]. The observations in literature concerning CCI’s predictive value on mortality after hip fractures vary. There are studies that confirm its significant correlation [5, 9, 24] and there are others that do not [25]. Accordingly preexisting dementia was often found to be an independent relevant influential factor [9, 13], whereas other studies did not show significant influence after correction for confounders [11, 26]. Sarcopenia is often part of the assessment variable “frailty” or “malnutrition” and also has been shown to impact mortality after hip fractures [5, 27].

Concerning POR prior to admission and CL, literature supports the correlation between higher care level [4], respective living in a nursing home [5, 6] and higher mortality. Fall associated death risk is rarely assessed in literature, e.g., Folbert et al. found no relevance in their univariate analysis [5]. After adjustment an insignificant tendency of lower survival is displayed in our analysis for lower need for care, home dwellers and *less* frequent fallers. This might on one hand be explained by the adjustment itself and, therefore, the revealed dependency of CL, POR and frequent falling by, e.g., PMS and BI. On the other hand, due to incomplete assessment of each parameter, this could have had a biasing effect as only cases could have been taken into account that were assessed fully in every of the ten included parameters. It is important to understand that patients living in nursing homes, attending care level 3 prior to admission and frequent fallers did not end up with higher survival rates than those with no care level, home dwellers and no or less falls in their history, they had in fact statistically inferior survival rates. However, it has to be supposed that maybe CL, POR and frequent falling are more dependent variables just as the previous mentioned that lost their significance in the multivariate analysis.

Comparisons between studies are difficult, because adjustment is performed through different parameters and not standardized. It can be assumed that especially taking into account the existing literature all significant variables for their own in the univariate analysis can be seen as predictive markers.

LOS and preexisting depression did not show any significant correlation to mortality already in the univariate analysis. A longer hospital stay was even associated to tendentially higher survival. As shown in this analysis discharge to rehabilitation or nursing homes increased LOS significantly for previous home dwellers, which revealed a significant confounding effect. Therefore, its effect on mortality becomes more indistinct. Heyes et al. registered slight increases in 1-year mortality after hip fracture for every subsequent week of inhospital stay without significance [28]. In a short term outcome analysis of 30-day mortality after hip fractures a significant impact of longer stay (11–14 days and over 14 days both compared to 1–5 days) and worse survival was observed [29]. Contrarily Yoo et al. observed increased 1-year mortality for patients staying 10 days in hospital or less [30]. Here once again literature is controversial which can result from different end points, included parameters, cohort diversity and a confounding effect of discharge accommodation as seen in this analysis. It has *also* to be assumed that LOS is influenced, furthermore, by reason to admission, POR, preexisting mobility, function and comorbidities.

Depression was found to be a significant risk factor for higher mortality in a meta-analysis [31] and in another study in combination with dementia even being a more hazardous risk factor [32]. Nevertheless, this investigation could not reveal according correlations.

### Limitations and strengths

We could examine a sufficient case number of orthogeriatric co-managed patients to provide a valid statistical evaluation. Important geriatric assessment parameters were included. Nevertheless, not every possible variable that could have affected mortality was considered, as especially in-hospital complications were missing in our analysis. These are known to have significant relevance for orthogeriatric patients’ survival [33]. Here an actual detailed analysis*,* which would go beyond the scope of this article, is in publication. Literature often makes use of the ASA Score which is not integrated in the actual investigation. The patients’ muscle strength could also not be taken into account as another predictive parameter due to missing of appropriate measurements. We did not consider different fracture types and the respective predictive importance of each parameter. A stratification of the ten variables included in the analysis could not be performed fracturewise due to too few remaining events in the respective fracture groups. A more rudimentary fracturewise stratification was performed in a previous publication. We, therefore, again have to emphasize our analysis as being an overview including a mean orthogeriatric patient cohort suffering from different injuries and causes for admission. It has to be taken into account that some injuries impact mortality more heavily (e.g., hip fractures) than others (e.g., forearm fractures) [34]. The group POR, LOS and GDS were excluded in the multivariate analysis, for not having shown univariate significance. Although this was also the case for gender, it was retained in the analysis for this factor to be known to have some impact on mortality considering the existing literature. To be able to deliver a valid multivariate analysis, ten events per variate included to the analysis are required to obtain valid results [18]. Because of the lack of data in several assessment parameters the number of cases that could be included into the Cox-regression analysis was reduced to 381 including 130 events. This still granted at least 10 events per involved variable. Still there was a risk of biasing by having excluded 280 cases. The diversity of the observed cohorts, the different end points (30-day, 1-year, 5-year mortality etc.) and the variety of parameters that are included in the analyses complicate comparisons to literature. Even though there are consensual recommendations for orthogeriatric outcome parameters, general and consistent adoption may be difficult in practice.

## Conclusion

In a variety of orthogeriatric co-managed patients we could identify age, prehospital PMS and BI on discharge as strong independent indicators of mortality after 2 years. Univariate significant correlations were also found for CCI, preexisting dementia, sarcopenia, frequent falls, living in nursing homes and higher care level, which all disappeared after adjustment. A high interdependency of these parameters with age, PMS and BI can consequently be supposed. All mentioned parameters qualify as predictors on their own. This result proposes setting higher emphasis on regaining self-support during the inward course as well as the significant relevance of preexisting mobility on the orthogeriatric patient’s outcome.

## Data Availability

The data sets used and analysed during the current study are available from the corresponding author on reasonable request.

## Abbreviations

ADL: Activities of daily living
AL: Assisted living
ASA: American Society of Anesthesiologists
BI: Barthel Index
CCI: Charlson-Comorbidity Index
CL: Care level
GDS: Geriatric depression scale
HR: Hazard ratio
LOS: Length of stay
MMSE: Mini-Mental-Status Examination
NH: Nursing home
PMS: Parker-Mobility Score
POR: Place of residence
PV: Private home
